# Hemato- Oncology Care in COVID-19 Pandemic: Crisis within a Crisis 

**DOI:** 10.31557/APJCP.2020.21.5.1173

**Published:** 2020-05

**Authors:** Tulika Seth, Abhishek Shankar, Shubham Roy, Deepak Sain

**Affiliations:** 1 *Department of Clinical Hematology, All India Institute of Medical Sciences, Delhi, India. *; 2 *Department of Radiation Oncology, Lady Hardinge Medical College & SSK Hospital, Delhi, India. *; 3 *Department of Pediatrics, Sitaram Bhartia Institute of Science and Research, Delhi, India. *; 4 *Cancer Control and Prevention Division, Indian Society of Clinical Oncology, Delhi, India. *

Taking care of Hemato-oncology patients in India, is one of the most resource intensive of all medical therapy. This takes up the maximum inpatient bed load, healthcare provider time and blood component resources of any hospital. The armamentarium necessary for management, includes all types of blood components, antibiotics, antifungals, chemotherapy; well trained staff (doctors, laboratory staff, nurses, counselors); financial, and social work support; all of which are often can be used in short supply. The lack of beds in most health facilities lead to admission delays, and over utilization of available space, thereby, increasing infection rates. 

The Coronavirus Disease-19 (COVID-19) pandemic, and subsequent national lockdown has severely affected the continuum of care. In a study from China, of COVID-19 positive patients (n=1,590), cancer patients were found to have a higher risk of severe adverse events compared with patients without cancer ([39%] vs [8%]; p=0·0003) (Shankar et al., 2020). 

Guidelines recommend rational rationing of resources (Emanuel et al., 2020; von Lilienfeld-Toal et al., 2020). In practice these are difficult and complex to implement. COVID-19 positive cases amongst those with new diagnosis of leukemia or other immune-compromised states with pre-existing lymphopenia, or prolonged neutropenia, will have a further increased risk of pneumonia, Acute Respiratory Distress Syndrome (ARDS) and prolonged viral shedding (Hirsch et al., 2013; Lehners et al., 2016). It is important to note that lymphopenia is the most important risk factor for pneumonia and mortality of COVID-19 patients. The coronavirus infection appears to cause lymphopenia, hence patients with pre-existing severe lymphopenia (<0.2x10^9^/L) will be at risk for rapid progression and severity of course (Hirsch et al., 2013; Lehners et al., 2016). Cancer patients and immune-compromised patients will also continue to shed the virus for a longer period of time, this is extrapolated from data from Severe Acute Respiratory syndrome (SARS) and influenza (Hirsch et al., 2013; Lehners et al., 2016). Hematopoietic stem cell transplant (HSCT) patients are at particular risk due to their prolonged and deep immune-compromised state. Hence non -essential HSCT are recommended to be postponed during the pandemic period. A risk being highlighted in the news and other media in many countries is the increased risk of acquisition of the infection from other patients or even infected health care providers. 

Most of our acute leukemia patients present with fever, cough and, pneumonia. Many already have pre-existing infections when they are diagnosed. But, if not treated, these patients may soon die from their disease itself. At present, only new cases of acute leukemia or high-risk myelodysplastic syndrome patients are getting admitted for treatment. If patients have radiological findings or influenza like symptoms, COVID testing is recommended prior to intensive chemotherapy to safe guard patients and minimize risk to the staff. Induction chemotherapy is necessary and cannot be postponed. Patients are being counseled regarding increased risk of mortality due to their disease as current experience shows that any co-existing disease increases mortality in COVID-19 patients, this is compounded by blood and platelet shortages, limited ICU beds, shortages of antibiotics etc. in the hospital due to the lockdown (Emanuel et al., 2020). Acute myeloid leukemia patients should be treated in isolation rooms with excellent barrier nursing, with strict limits on visitors to reduce the risk of acquiring coronavirus infection during intensive chemotherapy. These patients need blood components like platelet apheresis, packed red blood cells and acute promyelocytic patients will need fresh frozen plasma and cryoprecipitate. 

Numerous challenges are being faced by diagnosed patients who are already on therapy. If the patient has disease in remission, or post hematopoietic stem cell transplant and can safely defer any follow up or therapy to decrease their risk of getting COVID-19; by avoiding hospital visits. However, this is resulting in confusion and repeated questions from patients because of different instructions which somewhat deviate from the standard instructions given earlier. 

Guidelines for patients on subsequent courses of chemotherapy for acute lymphoblastic leukemia for pediatric and young adults is unchanged, as the excellent survival should not be compromised. In older patients, dose modifications may be done if previous cytopenias occurred or are anticipated .

Chronic Myeloid Leukemia (CML) therapy should also continue as per the pre COVID era. Patients should be advised to be compliant with their medicines and if stable can defer any upcoming molecular monitoring of BCR-ABL till a safer time. This will reduce the risk of infections and, therefore, treatment interruptions, which can cause progression to CML blast crisis, which at this point of time will be difficult to manage. The assessment of CML patients on wait and watch can be deferred. Patients can be monitored and treated with Intravenous Immunoglobulin (IVIG) and less cytotoxic drug Ibrutinib, where therapy cant be delayed. 

High risk myelodysplastic syndrome patients are immune-compromised due to their disease, hence therapy should not be delayed. These patients can be safely monitored and can receive Azacytadine at peripheral cancer clinics. They can also be advised erythropoietin or Thrombopoietin (TPO) receptor agonists to help decrease the need of frequent transfusion, which may be difficult. As much as possible transfusions should be taken close to the patients’ home. Prior to this crisis, patients would often travel across several State lines to receive transfusions at the tertiary care centers. Myeloproliferative neoplasm patients like primary myelofibrosis and polycythemia vera, again can be advised to continue medicines and be monitored via telemedicine. Patients on Ruxolitinib should be recommended to continue their medicine; cytopenia can occur in the initial 3 months of therapy and follow up by telemedicine and transfusion or dose adjustment for thrombocytopenia may be necessary. In patients of polycythemia vera, phlebotomy is a simple and important procedure and can be performed at district level hospitals.However, in case this is not possible, regular complete blood counts can be performed, then the patient can be put on hydroxyurea and monitored.

Multiple myeloma auto transplantation can be deferred if the patient is stable, though some relapsed patients may require urgent treatment. Telemedicine can be appropriately utilized to monitor patients and advise continuation of medicines in patients who are on maintenance therapy. Regular laboratory monitoring at a local lab can facilitate this care and increase the confidence and comfort levels of care in both the doctor and the patient. Initial therapy can be stated with bortezomib, lenalidomide and dexamethasone (RVD). For multiple myeloma maintenance either RVD, continuing only Rd (reduction of dexamethasone dose to 20 mg or even 10 mg) in elderly patients. 

Hodgkins Lymphoma (HL) patients should receive full curative therapy, and avoidance of delays or modifications should be done as much as possible to ensure adequate chances of excellent cure rates. There has been some debate of whether Bleomycin should be omitted, but there is no consensus on this strategy. For early stage HL patients, reducing chemotherapy cycles and adding radiotherapy has been recommended, centers should see the situation of the pandemic and feasibility of coming daily for radiation therapy. Oncologists should decide to postpone, discontinue or modify radiation therapy for the patients undergoing or planned for it, keeping an individualized risk/benefit assessment in mind (Shankar et al., 2020).

Aggressive non hodgkins lymphoma patients like Burkitts, Diffuse large B cell etc. need urgent and standard therapy. There should be no compromise in dose intensity or medicines. Patients of indolent lymphoma can be recommended to review after the pandemic has subsided and can be on follow up and supportive care. Telemedicine practice has been approved, but refilling cancer medicines online is not approved in the new guidelines (Mareš, 2016). Patients need to get a physical copy, though faxed or emailed prescriptions are now being allowed. This is a new process and every day poses new challenges to providers and patients. Even if patients are able to get prescriptions from us through daycare, Whatsapp or via email- many patients are unable to obtain medicines due to distribution problems, particularly in smaller towns. Teleconsultations are becoming an important tool to minimize disease related anxiety and stress among patients during this pandemic. Counselling by various doctors, nurses and social workers is now not encouraged to prevent the spread of infection. 

Delay or reducing intensity of therapy in patients of acute or chronic leukemias or myeloma patients (von Lilienfeld-Toal et al., 2020) seems the right thing to do and is being advised by many society guidelines. Patients on follow up have to postpone checkups, miss long and difficult to obtain appointments for PET scan and other tests which may result in missing early relapse cases. 

Fortunately, as most hemato-oncology patients routinely wear masks, follow good hygiene practices and are advised to not visit crowded places. The interruptions in routine care have had an undesirable and tragic consequence on the outcome of non-COVID patients. There is a multi- fold increase in emergency room visits in Hemato-oncology cases. The cost of this pandemic on both hemato-oncology patients and doctors (Mareš, 2016) is high. This pandemic is going to have long term serious consequences, and the oncologists must prepare for that in the coming time.

## References

[B1] American Society of Hematology. COVID-19 Resources. [Online].

[B2] Emanuel EJ, Persad G, Upshur R (2020). Fair allocation of scarce medical resources in the time of Covid-19. N Engl J Med.

[B3] Hirsch HH, Martino R, Ward KN (2013). Fourth European Conference on Infections in Leukaemia (ECIL-4): guidelines for diagnosis and treatment of human respiratory syncytial virus, parainfluenza virus, metapneumovirus, rhinovirus, and coronavirus. Clin Infect Dis.

[B4] Lehners N, Tabatabai J, Prifert C (2016). Long-Term Shedding of Influenza Virus, Parainfluenza Virus, Respiratory Syncytial Virus and Nosocomial Epidemiology in Patients with Hematological Disorders. PLoS One.

[B5] Mareš J (2016). Moral distress: Terminology, theories and models. Kontakt.

[B6] Shankar A, Saini D, Bhandari R (2020). Lung cancer management challenges amidst COVID-19 pandemic: hope lives here. Lung Cancer Management.

[B7] Shankar A, Saini D, Roy S (2020). Cancer care delivery challenges amidst Coronavirus Disease - 19 (COVID-19) Outbreak: Specific precautions for cancer patients and cancer care providers to prevent spread. Asian Pac J Cancer Prev.

[B8] The European Society for Blood and Marrow Transplantation. Coronavirus disease COVI-19: EBMT recommendation update March 23, 2020 [Online].

[B9] von Lilienfeld-Toal M, Vehreschild JJ, Cornely O (2020). Frequently asked questions regarding SARS-CoV-2 in cancer patients-recommendations for clinicians caring for patients with malignant diseases. Leukemia.

[B10] World Health Organization (2020). Maintaining a safe and adequate blood supply during the pandemic outbreak of coronavirus disease (COVID-19). [Online].

